# Monopole directional antenna bioinspired in elliptical leaf with golden ratio for WLAN and 4G applications

**DOI:** 10.1038/s41598-022-21733-z

**Published:** 2022-11-04

**Authors:** Eduarda Froes, Paulo F. Silva Junior, Ewaldo E. C. Santana, Carlos M. Sousa Junior, Paulo H. F. Silva, Carlos A. M. Cruz, Vivianne S. Aquino, Luis S. O. Castro, Raimundo C. S. Freire, Mauro S. S. Pinto

**Affiliations:** 1 Graduating Program in Computation Engineering Systems, State University of Maranhão, São Luís, 65000-000 Brazil; 2 Graduating Program in Electrical Engineering, Federal Institute of Paraiba, João Pessoa, 58135-000 Brazil; 3 Graduating Program in Electrical Engineering, Federal University of Amazonas, Amazonas, 69460-000 Brazil; 4 Graduating Program in Electrical Engineering, Federal University of Campina Grande, Paraiba, 58490-900 Brazil

**Keywords:** Engineering, Electrical and electronic engineering

## Abstract

In this work, it is proposed the development a new monopole directional antenna, bioinspired in elliptical leaf, with cut by golden ratio, for 4G band application, by the use of the technique of the cut of the radiating element for the increasing of the antenna perimeter, being the first work to use this technique in a bioinspired antenna, promotes resonance frequency turned, and reconfiguring of the antenna parameters as bandwidth, radiation pattern and gain, with the use of the reflector near to the group plane, without the insertion of active devices as the pin diode or change in radiating element. The shape antenna is generated by Gielis formula, built in FR4 substrate, with cuts calculated by golden ratio. To compare the results of the bioinspired monopole on the elliptical sheet, a square-shaped monopole antenna was designed, simulated and measured, the structures were designed in the MATLAB software version 2015(b) and the simulations were performed in the Ansys software version 2016. In the results compared between the square monopole and the bioinspired antenna in the elliptical sheet, it can be seen that the measured bioinspired antenna, compared to the square monopole, presented a bandwidth reduction of 77.27%, a more compact structure, with a reduction of 98%, covering the wireless local area network, and long-time evolution 4G at 2.5 GHz. The proposed technique uses a reflector on the ground plane, to change the parameters of the monopole planar antenna, of omnidirectional radiation pattern to a directional, maintaining the characteristics of the broadband, half-power beamwidth great than 100°, with high current density, and similar gain of a directional antenna. From the results, it has been observed that the elliptical leaf monopole antenna shows broadband characteristics, with a half-power beamwidth of 128°, wideband, the bandwidth of 500 MHz, a gain of 6.28 dBi, a current density of 13.01 A/m^2^, and circular polarization.

## Introduction

Modern mobile communications require research into compact devices operating in several technologies. The antennas are important devices because they perform the receiver and transmission of signals, which should present a compact structure, low-cost, circular polarization, and wideband. Several techniques are used in antennas to modify the shape and parameters, including fractal and bioinspired technologies^1–5^.

A planar monopole antenna is also known as a monopole antenna, as characterized by presented wideband an ultra-wideband, compact structure, low gain, operating in several frequency bands, numerous shapes, and omnidirectional radiation pattern^7,8^. Some techniques were used to change monopole antenna parameters, such as modifications in the ground plane^9^, the use of the metamaterials^10^, and the insertion of the reflector in the antenna back^4^.

Bioinspired antennas are devices that use shapes of living beings to alter some antenna response parameters and are used in several applications, such as narrow-band, wideband, ultra-wideband, wireless local area network, wireless fidelity, industrial, scientific and medical-ISM, mobile communications, and manifold antenna types, including, patch, planar monopole, aperture, etc.^1–5^. The shapes of the radiating elements of the bioinspired antennas can be generated by several methods, such as drawing in simulator software, and images generated by equations^2,4,5,11–13^. With the use of bioinspired shapes in the antenna, technology is possible for generating compact structures with large perimeters and developed small electromagnetic devices operating at low frequencies^2^.

The Gielis formula is a polar equation capable of generating several forms, including plant forms^11–13^. Plants and other organisms produce energy through the process of photosynthesis, in which they harvest light, transforming captured electromagnetic energy into chemical energy to sustain life. The plant has a complex light-harvesting system, and a reaction centre^14,15^, acting as a receiving antenna. Thus, the shapes of plants are optimized for the light-harvesting, an electromagnetic wave, in the THz range, their shapes have a compact structure with a greater perimeter, an important feature in antenna technology since the resonance frequency of an antenna is related to the perimeter of the radiating element in a patch antenna^4–7^.

Several works use the Gielis formula in monopole antenna shape development. In Ref.^2^ was developed, a monopole planar antenna, bioinspired in *Opuntia ficus-indica* plant-shape, with hybrid feed, operating in wireless local area network (WLAN) at 5 GHz. The technique of use of a reflector to the change of the bandwidth and increasing of gain in bioinspired antennas is introduced by Ref.^4^, with the development of a directive monopole antenna bioinspired in the sugar-cane plant for operation in 4G in 700 MHz, presented more compact structure, reduction of 95% compared with the square shape, bandwidth of 165 MHz, and a maximum gain of 7.7 dBi. In Ref.^5^ was used, the bioinspired shape of a monopole antenna bioinspired in *Ginkgo biloba* leaf, built in textile material and laminate cooper, covering 2G, 3G, and 4G bands, with a bandwidth of 2.70 GHz. In Ref.^6^ was used the bioinspired shape in the development of dielectric resonator antenna for wideband sub-6 GHz range, generated by Gielis formula, operating in 5.5 GHz and maximum gain of 5 dBi. Some bioinspired antenna shapes as developed by Gielis formula in Refs.^11,16^, with devices built in FR4 and denim, operating in ultra-wideband, 2G, 3G, 4G, and wireless local area network (WLAN) range. A monopole antenna is bioinspired on the *Acer macrophyllum* leaf for monitoring in circuit breaker monitoring at 230 kV, as the non-invasive solution is developed in Ref.^17^.

In this work is developed a new monopole directional antenna, bioinspired in elliptical leaf, with aperture by golden ratio for WLAN and long-time evolution (LTE) 4G applications at 2.5 GHz, by the use of the technique of the cut of the radiating element for the increasing of the antenna perimeter, promotes resonance frequency turned, and reconfiguring of the antenna parameters as bandwidth, radiation pattern and gain, with the use of the reflector near to the group plane, without the insertion of active devices as the pin diode or change in radiating element. This paper is divided in three sections besides of this introduction. In “Materials and methods” are presented the materials and methods used in the development of the work. In “Results and discussions” are related the results and discussions, and in “Final considerations” the final considerations.

## Materials and methods

In this work is proposed the development of a directional monopole antenna, bioinspired in the elliptical leaf, generated by Gielis formula, with cut in the radiation element according the golden ratio, operating in the WLAN (2.4–2.483 GHz) LTE 4G band at 2.5 GHz (2.5–2.6 GHz).

In this work, it is proposed a new monopole antenna, bioinspired in an elliptical sheet, in which some techniques are applied. The first is the use of cuts in the radiating element in order to increase the perimeter of the antenna, something similar to what is done in antennas with fractal shapes, such as the Koch fractal, which can be positive, with the increase of the total structure, or by removal of parts of the radiating element, generating similar structures. The parts removed from the radiating element are similar to the original structure with reduced size, in which the gold number was used to calculate these parts, as it is a known factor and applied in several technologies, which proved to be effective for increasing the perimeter and consequent reduction of the resonant frequency of the antenna. The second technique was the use of a reflector close to the ground plane of the antenna, with which it was possible to change the bandwidth and gain of the antenna, keeping the half-power beamwidth and the current density constant, with circular polarization, thus, it was possible to reconfigure the antenna response inserting no other device into the antenna, such as a Pin diode, transmission lines, internal openings or any other technique.

The methodology to the development of bioinspired antennas used in this work is adapted of Ref.^7^, with a choice of the frequencies applications for WLAN and mobile communications in LTE 4G band, built in fibber glass (FR4) dielectric material, relative permittivity of *ε*_*r*_ = 4.4, loss tangent of tan(*δ*) = 0.02, thickness of 1.55 mm using shape of elliptical leaf, generated by MATLAB version 2015(b), simulated by Ansys software version 2016, and performed in the Laboratory of Measurements of the Federal Institute of Paraiba (IFPB), Campus of João Pessoa using a Vector Network Analyzer (VNA) of Agilent model S5071C (300 kHz–20 GHz).

### Monopole directional antenna

A Monopole antenna is a patch antenna with a truncated ground plane, broadband characteristics, reduced dimensions, and the omnidirectional radiation pattern used in ultra-wideband technology^18^. The match impedances between the transmission line and the radiating element are used techniques such as cut, and variation of the length of the ground plane^19,20^.

According to Ref.^19^ the design values of a planar monopole can be approximated from the perimeter (*p*) of antennas with circular and square shapes. According to Ref.^18^, the current distribution is more concentrated at the ends than at the centre of the radiating element, the distance travelled by the current influences the resonant frequency of the antenna, so increasing the antenna perimeter provides a reduction in the first resonant frequency (*f*_*r*_), which can be got by:

1 $$ {f_{r} ({\text{GHz}}) = \frac{{{300}}}{{p\sqrt {\varepsilon_{reff} } }}}, $$

were ${\varepsilon }_{{r}_{eff}}$ is the effective permittivity, given by Ref.^19^:

2 $$ \varepsilon_{reff} \approx \frac{{(\varepsilon_{r} + 1)}}{2}. $$

The directional planar monopole antenna proposed in this work uses a reflector in the back of the antenna, with a distance based on the wavelength at the antenna resonance frequency^4,20^. According to Ref.^20^ the proximity of the ground plane of the monopole of the conductor promotes modification of antenna parameters, such as bandwidth, gain, and half-power beamwidth. Thus, by the use of this technique is possible the reconfigurable the antenna parameters, changed the broadside to the end-fire propagation direction, increase the gain, and concentrate bandwidth.

### Gielis Formula

An observed difficulty between the design, simulation, and construction of a patch antenna is the differences between the design data, the simulation, and the construction of the antenna. An antenna design considered acceptable presents a difference that can vary between 1 and 5% of the values obtained considering the operating frequencies, bandwidth, and the applied technology. To reduce differences by implementing equations for automatic generation of radiating element shapes. A polar equation used for generating shapes observed in nature, for generating shapes for patch antennas is the Gielis formula^12,13^.

According to Refs.^12,13^ circular shapes, squares, ellipses and rectangles are members of the group of superellipses which have limited symmetry as a disadvantage. Using the polar coordinate, by substituting of $x = r\cos (\theta )$ and $y = r\sin (\theta )$, and entering the *m*/4 angle argument, introduces a specific rotational symmetry. The arguments ${n}_{i}$ and $m$, to the set of real numbers, and *a* and *b* are nonzero real numbers. Gielis formula can be used by multiplying by a function, forming a generic equation that generates a large class of super, and sub-forms, including the super and the sub-circle as a special case, similar structures to the generated by L-system and by fractals, Euclidean and non-Euclidean forms, such as those observed in nature. Gielis formula is giver by:

3 $$ r(\theta ) = \left[ {\left| {\frac{1}{a}\cos \left. {\left( {\frac{m}{4}\theta } \right)^{{n_{2} }} } \right| + \left| {\left. {\frac{1}{b}\sin \left( {\frac{m}{4}\theta } \right)} \right|^{{n_{3} }} } \right.} \right.} \right]^{{{\raise0.7ex\hbox{${ - 1}$} \!\mathord{\left/ {\vphantom {{ - 1} {n_{1} }}}\right.\kern-\nulldelimiterspace} \!\lower0.7ex\hbox{${n_{1} }$}}}} $$

### Golden Ratio

The golden ratio, is an irrational number represented by the symbol *ϕ*, *ϕ* = 1.6180339227498…, that must be obtained by Fibonacci numbers, it can be described by length of the two segments *a* and *b* whit *a* > *b* > 0, given by Refs.^21,22^:

4 $$\frac{a}{b}=\frac{a+b}{a}\to \phi =1+\frac{1}{\phi }\to \phi =1+\frac{1}{1+\phi }\to 1+\frac{1}{1+\frac{1}{1+\frac{1}{1+\cdots }}}\cong 1.6180\dots $$

where *ϕ* = *a/b*. A possible visualization of the golden ratio is approximated by Lucas Numbers, with the ratio of natural numbers (*n*) by division of a posterior number by the sum of predecessor numbers. Table 1 is shown the Lucas Numbers in rational and decimal representation.

**Table 1:** Lucas numbers.

*n*	Rational representation	Decimal value
0	1	1.00000
1	2/1	2.00000
2	(2 + 1)/2 = 3/2	1.50000
3	(3 + 2)/3 = 5/3	1.66666
4	(5 + 3)/5 = 8/5	1.60000
5	(8 + 5)/8 = 13/8	1.62500
6	(13 + 8)/13 = 21/13	1.61539
7	(21 + 13)/21 = 34/21	1.61905
8	(34 + 21)/34 = 55/34	1.61768
9	(55 + 34)/55 = 89/55	1.61878
10	(89 + 55)/89 = 144/89	1.61798

In this work, the golden ratio is used to calculate the dimension of the cuts in the elliptical leaf and increase the perimeter of the antennas to alter resonant frequency.

### Antenna design

**Figure 1:**
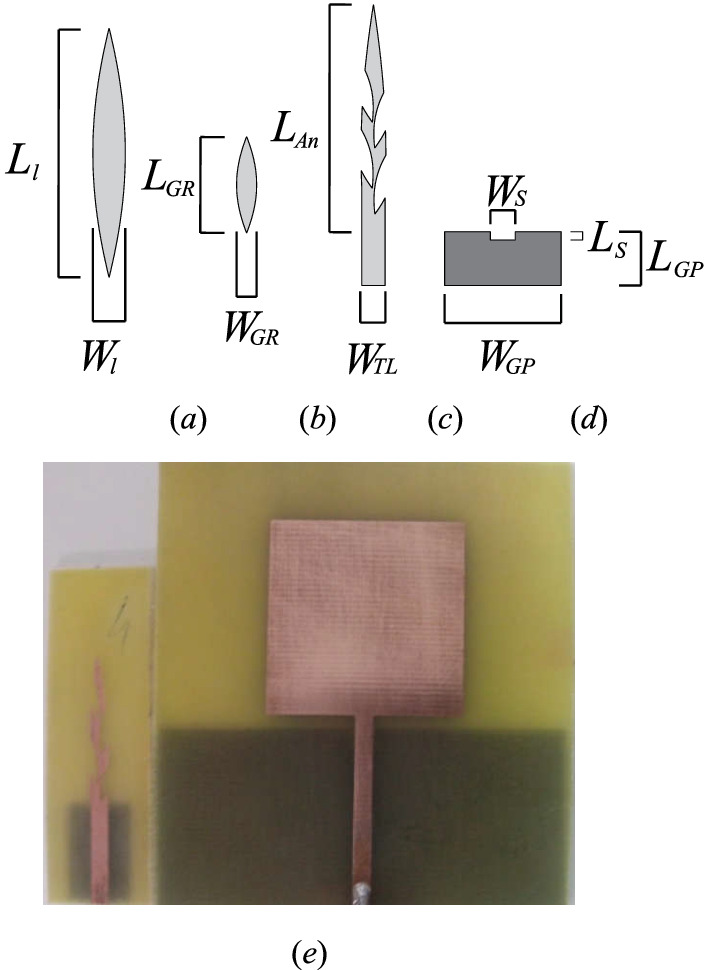
Project of directional monopole antenna bioinspired in the elliptical leaf: (**a**) Dimensions of the leaf performed in MATLAB 2015(b); (**b**) Dimensions of the leaf calculated by golden ratio performed in MATLAB 2015(b); (**c**) simulated antenna with the cuts performed in Ansys 2016; (**d**) ground plane of the antenna.

The project of the elliptical leaf bioinspired antenna, with the elliptical leaves, simulated and prototype antenna, can be observe in Fig. 1, and the values of the dimensions in Table 2. In the first step, it is used the perimeter of the Euclidean structure as initial values, with values of the square antenna side (*L*_*An*_ = *W*_*An*_), transmission line width (*W*_*Tl*_), transmission line length (*L*_*Tl*_), ground plane width (*W*_*GP*_), ground plane length (*L*_*GP*_)and the length (*L*_*S*_) and width (*W*_*S*_) of the slit on the ground plane (LS). In work were use the dimension of the square monopole antenna by the Eq. (1). The elliptical leaf, Fig. 1a, for dimensions of length (*L*_*l*_) and width (*W*_*An*_) of the leaf, were generated by Gielis formula in the MATLAB software version 2015(b) with the parameters of *m* = 2, *n*_*1*_ = 400, *n*_*2*_ = 1200, *n*_*3*_ = 1200, *a* = 1, *b* = 1. The values of *x*, *y*, and *z* axis are adjusted by the A = 36.16, circle angle step of H = 360, the range of *f*_*i*_ = [0:π/(H):2π]. The code MATLAB version 2015(b) used in the elliptical leaf is:

$$ x = \, \left( {r. \times {\text{cos}}\left( {f_{i} } \right)} \right); $$

$$ x = x./{\text{max}}\left( {{\text{abs}}\left( x \right)} \right)./{3}; $$

$$ y = r.*{\text{sin}}\left( {f_{i} } \right); $$

$$ y = y./{\text{max}}\left( {{\text{abs}}\left( y \right)} \right); $$

$$ z = {\text{ zeros}}\left( {{\text{size}}\left( y \right)} \right); $$

$$ x = {\text{A}}.*\left( {0.{125}.*x} \right)^{\prime}; $$

$$ y = A.*y^{\prime}.*0.{318}; $$

$$ z = {\text{ A}}.*z^{\prime}. $$

**Table 2:** Dimensions of the monopole antennas (mm).

Antenna	*L*_*l*_	*L*_*An*_	*W*_*l*_	*L*_*GP*_	*W*_*GP*_	*L*_*S*_	*W*_*S*_	*L*_*TL*_	*W*_*TL*_
Elliptical leaf	23.00	36.16	3.00	15.00	12.00	1.00	3.00	16.00	2.80
Square shape		29.00		28.00	60.00	2.00	3.00	31.00	2.80

The image is exported in DXF formatted and imported in the Ansys software version 2016.

Figure 1b shows the elliptical leaf calculated by the golden ratio, used in the cut of elliptical leaf antenna, for the increase in the perimeter, and reduction of the resonance frequency. The position of the leaves was used an angle inclination of 16°, with a distance of 3 mm from the ground plane to the right leaf and 6 mm to the second leaf, to the leaf of the on the left side was used 6 mm distance between the ground plane and the first leaf on the left and 9 mm for the second leaf. The structure as calculated for dimension of length (*L*_*GR*_) and width (*W*_*GR*_), applied the golden ratio in the length and width of the elliptical antenna, with two interaction of *L*_*An*_, and one to *W*_*An*_, keeping the regular shape between length and width, see Fig. 1c.

$$ {1}^{ \circ } L{-}{\text{ Interaction}}\,{\text{for}}\,L_{An} = { 23}\,{\text{mm}}, $$

$$ {2}^{ \circ } L{-}{\text{ Interaction}}\,{\text{for}}\,{\text{L}}_{An} = { 14}.{2}\,{\text{mm,}} $$

$$ {1}^{ \circ } W_{{}} {-}{\text{ Interaction}}\,{\text{for}}\,W_{l} = { 3}\,{\text{mm}}, $$

The calculated transmission line width (*W*_*LT*_) was 2.8 mm, the width of the slit on the ground plane (*W*_*S*_) with the best result was 3 mm, and the length of the slit on the ground plane (*L*_*S*_) was 1 mm for bioinspired antenna, and 2 mm to the square monopole, see Fig. 1d. Figure 1e shows the prototypes of the square and elliptical leaf with the cut of the golden ratio monopole antennas, which can be compared the dimensions of the structures.

The directional monopole antenna design uses a copper reflector plate with dimensions:

- A length of 130 mm, a width of 130 mm, and a distance of 60 mm from the square monopole;
- A length of 44 mm, a width of 22 mm, and 20 mm of the distance of the bioinspired monopole in the elliptical leaf.

The reflector distance of the antennas is calculated by wavelength (*λ*_0_), with *λ*_0_/4 to the bioinspired antenna and *λ*_0_/2 to the square shape monopole antenna. According to Ref.^20^ the distance of a fraction of wavelength, near the ground plane of the monopole antenna, change some parameters of the antenna, such as radiation pattern, gain, and bandwidth, which can be used in the project of the directional monopole bioinspired antenna.

## Results and discussions

An antenna is evaluated according to the results of its parameters, such as the reflection coefficient, given by parameter S11, in which the bandwidth, the resonance frequency and the best result in the loss of return can be observed. Other important parameters are the gain, given in dBi, which is the gain in dB compared to an isotropic antenna, the half power beamwidth, with the indication of the radiation angle of the antenna in degrees, the current density on the surface of the structure, and the axial ratio, in which the type of antenna polarization can be identified.

The S_11_ parameter is the reflection coefficient of port 1, the power level that is perceived by port 1 from port 1 in dB. Thus, the lower the value, the more power is radiated by the antenna and the lower the power returned to the port. In the evaluation of the antenna bandwidth, values below − 10 dB are considered, which shows that the matching of the impedances of the transmission line and the radiating element guarantees that 90% of the power is being used by the antenna.

**Figure 2:**
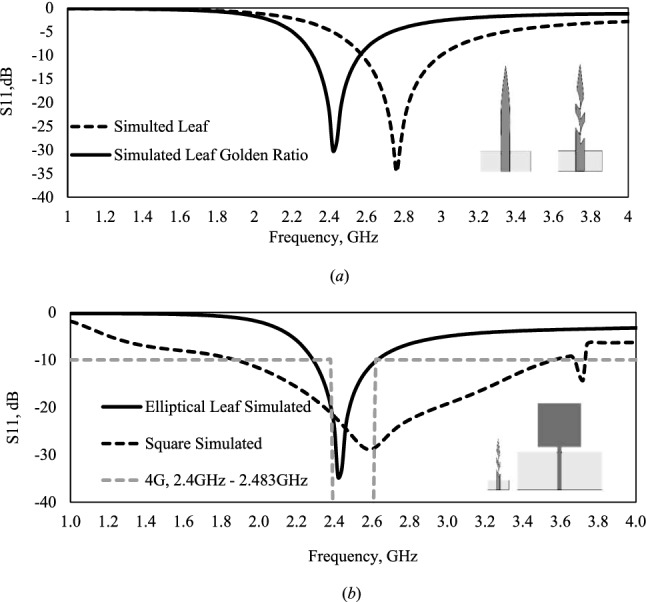
Comparison of simulated square shape and elliptical leaf directional monopole antennas performed in Ansys 2016: (**a**) Elliptical leaf, and elliptical leaf with aperture by the golden ration without the reflector; (**b**) square shape and elliptical leaf with aperture by the golden ratio with reflector.

Figure 2 shows the comparison of the simulations of the leaf monopole antenna, the antenna with the aperture performed by the golden ratio without the reflector, and the simulation of the square shape and the elliptical leaf directional monopole antenna with the reflector. The aperture performed by the golden ratio increased the perimeter of the monopole antenna, with the changed the resonance frequency, 2.76 GHz for the leaf, and 2.42 GHz for the leaf with the aperture by the golden ratio, a variation of 12.32%. In the results, it can be observed an increase of the bandwidth of 160 MHz, with 240 MHz for the leaf, and 400 MHz for the leaf with the aperture by the golden ratio Fig. 2a.

The comparison of simulated S_11_ parameter of the square shape and bioinspired leaf monopole antenna with aperture by golden ratio with reflector can be visualized in Fig. 2b. The square monopole antenna shows result of − 10 dB in the frequencies of 1.88–3.58 GHz, with a bandwidth of 1.70 GHz, and a central resonance frequency at 2.62 GHz. The elliptical leaf got results of − 10 dB in the frequencies of 2.30–2.62 GHz, with a bandwidth of 320 MHz, and a central resonance frequency at 2.42 GHz. In the comparison of the square monopole, the bioinspired elliptical leaf directional monopole antenna shows a reduction of 81.18% in the bandwidth, covering the 4G band at 2.4 GHz.

An important parameter is the bandwidth, in the frequency range between − 10 dB, it shows that there is a matching of impedances between the transmission line and the radiating element, guaranteeing the minimum power returned to the gate and the maximum power radiated by the antenna. In the result, the central resonance frequency can be observed, which must present the minimum return loss. The greater the bandwidth, the greater the amount of data that can be sent or received by the antenna, and for each technology, there is an operating bandwidth and the antennas must operate within the required level.

**Figure 3:**
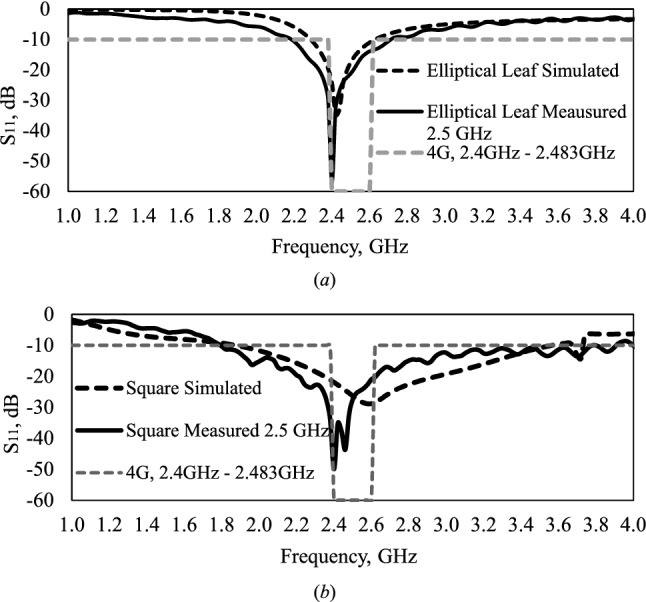
Comparison of simulated performed in Ansys 2016 and measured results of directional monopole antennas: (**a**) elliptical leaf; (**b**) square shape.

A comparison between of the S_11_ parameters of simulated and measured results of square shape and leaf elliptical directional monopole antennas can be observed in Fig. 3. From de results is possible observed that the measured antenna presents a bandwidth of 500 MHz, 34% greater than that of the simulation, with resonance frequency at 2.40 GHz, a difference from 0.88%. The difference in the bandwidth and resonance frequency can be attributed to variations in the dielectric structure, but we notice that the result covers the 4G band at 2.40 GHz, Fig. 3a. The square monopole antenna obtained close values of bandwidth, simulated at 1.70 GHz, and measured at 2.20 GHz, with resonance frequencies of 2.58 GHz and 2.40 GHz, a difference of 1.65%, Fig. 3b.

**Figure 4:**
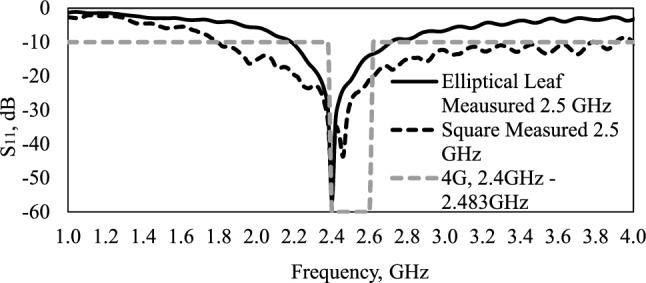
Comparison of measured results of square and elliptical leaf directional monopole antenna.

Figure 4 shows the comparison between measured S_11_ parameters of the square shape and leaf elliptical directional monopole antennas, and the values in Table 3. We noticed the elliptical leaf monopole antenna presents a more concentrated bandwidth covering the 4G band, at 2.4 GHz, and a greater loss return, less than − 56 dB, in a more compact structure. Considering the reflector and monopole antenna area, the square directional monopole presents a total area of 1039.35 cm^3^, and an elliptical leaf monopole of 20.81 cm^3^, thus, the bioinspired antenna presents a reduction of 98%.

**Table 3:** Results of measured monopole antennas.

Antenna	BW (MHz)	*f*_1_ (GHz)	*f*_2_ (GHz)	*f*_0_ (GHz)	Gain (dBi)
Elliptical leaf	500	2.20	2.70	2.40	6.28
Square	2240	1.80	4.04	2.46	7.53

**Figure 5:**
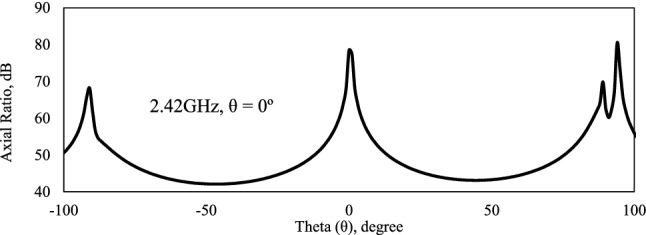
Simulation axial ratio of elliptical leaf directional monopole antenna in Ansys 2016.

The axial ratio shows the circular polarization of an antenna, indicating that it can receive or send a signal at any position, with the transmission coefficient does not depend on the device position. It is defined as the ratio between the major and minor axis of a circularly polarized antenna pattern. If an antenna has perfect circular polarization, then this ratio would be 1 (0 dB). Figure 5 shows the simulated results of the axial ratio of the leaf elliptical directional monopole antenna. An antenna with circular polarization is made up of two orthogonal electric field components of equal amplitude and 90° out of phase, the closer the axial ratio is to 0 dB, the better^23^. The result indicates the circular polarization of the bioinspired antenna, com maximum gain in dB, at theta angle, θ = 0°. An antenna with circular polarization must be received or transmitted a signal in any angular position.

**Figure 6:**
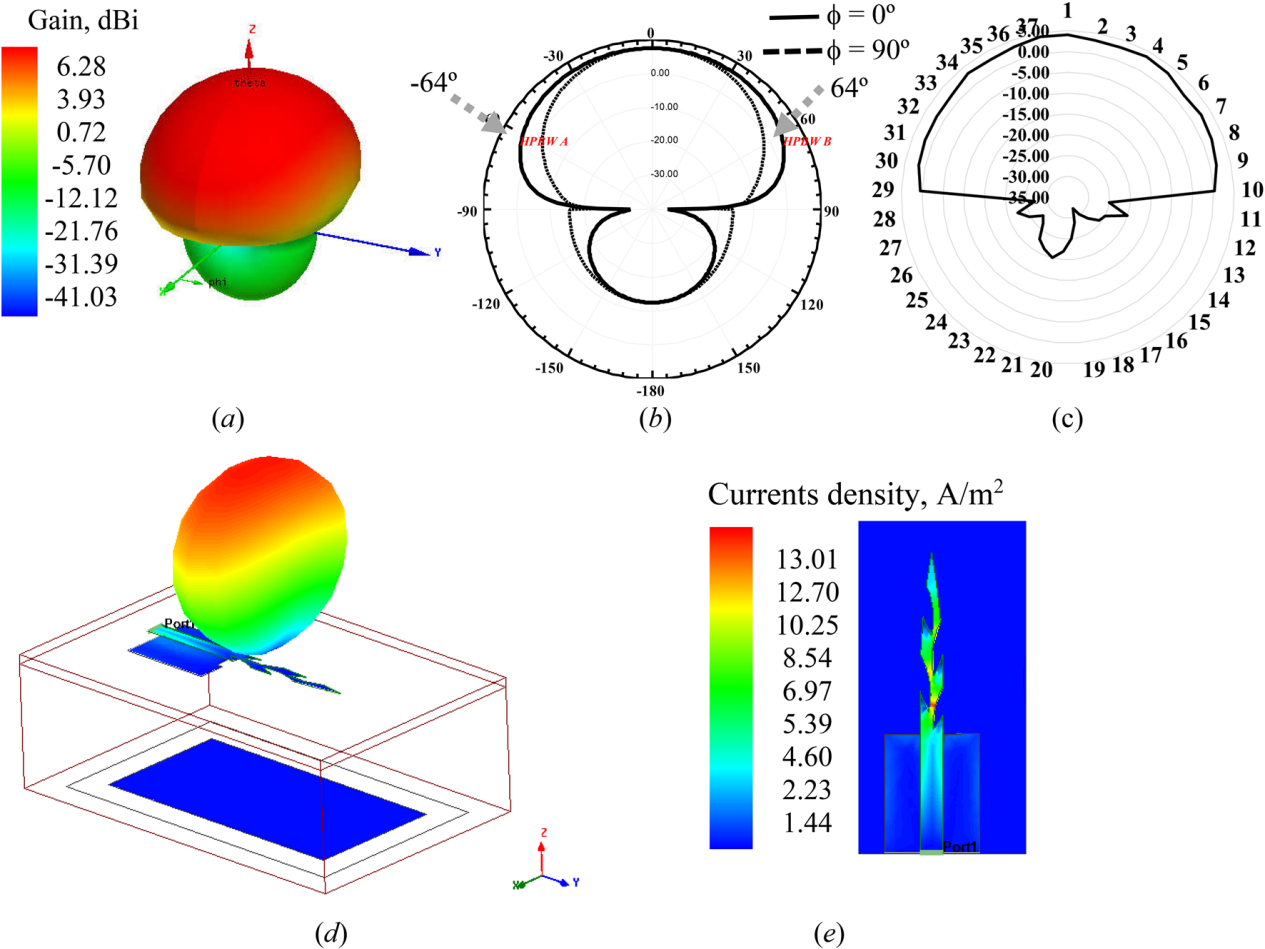
Radiation pattern of elliptical leaf directional monopole antenna: (**a**) Simulated gain, 3D performed in Ansys 2016; (**b**) Simulated HPBW, 2D plan-E and plan-H performed in Ansys 2016; (**c**) Measured radiation patter of the bioinspired monopole with reflector; (**d**) Simulated radiation patter of the bioinspired monopole with reflector performed in Ansys 2016; (**e**) Simulated current density with monopole antenna performed in Ansys 2016.

Radiation patterns inform the antenna parameters of the electric field, plane E, and magnetic field, plan H. The direction of propagation of the electromagnetic wave is shown by plane E, with the half-power beamwidth (HPBW) mark. Figure 6 shows the simulated and measured results of radiation pattern and current density of the leaf elliptical directional monopole antenna, and value of the gain in Table 3. The square and elliptical leaf monopole antennas show close results of the gain (Fig. 6a), HPBW of elliptical monopole antenna 128° in the plane-E, presents similar gain of the end-fire radiation pattern, HPBW of the broadside type (Fig. 6b), similar measured radiation pattern (Fig. 6c), and high current density, 13.01 A/m^2^ (Fig. 6e). Thus, from the use of the technique is possible a result directional in an omnidirectional antenna, with wideband characteristics in the compact structure.

The proposed directional monopole bioinspired antenna used the shape of the elliptical leaf used two techniques in the development of the project. First is the cuts in the radiating element the similar shapes of the elliptical leaf reduced, calculated by golden ratio, for the increasing of the antenna perimeter for turner of the resonance frequency, being the first work to use this technique in a bioinspired antenna. The second technique use a reflector on the ground plane for change the antenna parameters, was observed in Ref.^20^ and used in the monopole antenna bioinspired in plants in Ref.^4^, with the evaluation of the change of bandwidth, current density, and gain.

The comparison of the results of the directional monopole antennas bioinspired elliptical leaf and sugar-cane^4^ can be observed in Table 4. The elliptical leaf shows bandwidth 203%, and current density 130.67% greater of sugar-cane antenna. The current density of elliptical is related to the less dimensions of the sugar-cane, and greater heat dissipation by the Joule effect. The greatest gain observed in sugarcane is due to the greater amount of metal in the radiating element.

**Table 4:** Comparison of the directional monopole antennas bioinspired elliptical leaf and sugar-cane^4^.

Antenna	BW (MHz)	HPBW	Gain (dBi)	Current density (A/m^2^)
Elliptical leaf (2.5 GHz)	500	128°	6.28	13.01
Sugar-cane (700 MHz)	165	120°	7.7	5.64

A proposal for future work is the use of meta-deflectors, which can gain in the reflection of the antenna radiation at the phase angle without compromising other propagation characteristics^24^, and the metasurfaces, with which it is possible to develop structures with better wave reflection, considering the polarization designed for the antenna, as stated in the research of Ref.^25^.

## Final considerations

In this paper, it was developed a new directional monopole antenna bioinspired in elliptical leaf, with cut calculated by the golden ratio, in low-cost material, FR4, generated by the Gielis formula, applied to WLAN and 4G band at 2.5 GHz. Two techniques are applied in the development of the new antenna, the cut of the element radiating by the golden ratio, for the increasing of the antenna perimeter, and turned of the resonance frequency, and the directional technique used a conductor plate next to the ground plane in a monopole antenna, promoting changes in the antenna parameter, such as bandwidth, gain, and radiation pattern. The results of the bioinspired monopole antenna were compared with the square monopole antenna, and the bioinspired shape got a more compact structure with an area 98% less than the square shape, directional radiation pattern, the maximum gain of 6.28 dBi, circular polarization, and characteristic of the wideband antenna, with a bandwidth of 500 MHz, half-power beamwidth of 128°, covering the 4G band, at 2.5 GHz, and WLAN range, at 2.4 GHz. Using a reflector next to the ground plane monopole antenna was possible to get directional parameter characteristics in the omnidirectional antennas, without altering the monopole structure. This technique can be used in many applications.

## Data Availability

The datasets generated and/or analysed during the current study are available in the Data_Scientific Repor_Elliptical Leaf.rar repository, https://mega.nz/file/nhp3WQSA#IEndJpVjBQzty5__toFLqEemrVZG_rJ3aDKX3czepZA.
